# Coffee Consumption and Pancreatic Cancer Risk: A Meta-Epidemiological Study of Population-based Cohort Studies

**DOI:** 10.31557/APJCP.2020.21.9.2793

**Published:** 2020-09

**Authors:** Jong-Myon Bae, Sung Ryul Shim

**Affiliations:** 1 *Department of Preventive Medicine, Jeju National University College of Medicine, Jeju, Republic of Korea. *; 2 *Department of Preventive Medicine, Korea University College of Medicine, Seoul, Republic of Korea. *

**Keywords:** Coffee, pancreas neoplasm, cohort studies, meta-analysis

## Abstract

**Objective:**

Previous systematic reviews evaluating the association between coffee consumption and pancreatic cancer showed inconsistent results. The aim was to conduct a meta-epidemiological study to explore further the association between coffee consumption and the incidence of pancreatic cancer.

**Methods:**

The selection criteria were defined as a population-based prospective cohort study reporting adjusted relative risk (RR) and their 95% confidence interval (95%CI) of pancreatic cancer occurrence according to coffee consumption. Adjusted RR for the highest versus the lowest level of coffee consumption in each study was extracted. A fixed-effect model was applied to calculate a summary RR (sRR) and its 95%CI. Two-stage random-effects dose-response meta-analysis (DRMA) was performed to estimate the incidence risk per unit dose (cup per day).

**Results:**

Twelve cohort studies were selected for meta-analysis. The total number of cohort participants was 3,230,053, and pancreatic cancer incidents were 10,587. The sRR of pancreatic cancer risk for the highest versus the lowest level of coffee consumption indicated no statistical significance (sRR=0.98, 95% CI: 0.88-1.10; I-squared=0.0%). Two-stage random-effect DRMA showed the non-linear relationship between the amount of coffee consumption and pancreatic cancer risk. And the RR for an increment of one cup per day of coffee consumption was 0.97 (95%CI: 0.91-1.04, P=0.42), without statistically significant.

**Conclusion:**

There was no association between coffee consumption habits and pancreatic cancer risk. And there was no statistical significance in the dose-response relationship between the amount of coffee consumption and pancreatic cancer risk. Finding the turning point would be important because it can be critical information for the prevention of pancreatic cancer.

## Introduction

As pancreatic cancer is notorious for having a poor prognosis (Bray et al., 2018), finding modifiable risk factors is paramount. While coffee is a popular beverage globally, Zhao et al., (2020) concluded that ‘there is highly suggestive evidence for an inverse association between coffee intake and risk of liver and endometrial cancer.’ But Tsai and Chang (2019) concluded that ‘the association between coffee and pancreatic cancer is unclear.’ 

Table 1 summarizes the results of seven systematic reviews of observational studies that evaluated the association between coffee consumption and pancreatic cancer risk (Dong et al., 2011; Yu et al., 2011; Turati et al., 2012; Ran et al., 2016; Wang et al., 2016; Nie et al., 2016; Li et al., 2019). Summary relative risk (sRR) calculated for the highest versus the lowest level of coffee consumption were inconsistent. Because the directions of sRRs were conflicting, and statistical significance differed among systematic reviews. For these facts, Kawada (2019) suggested that “there is a need to present clear inclusion and exclusion criteria, according to a recommendation from the Cochrane Collaboration that high heterogeneity of the results should be avoided.” The authors accept his suggestion for the following three reasons. First, among the papers selected from the previous systematic reviews, cohort studies following pancreatic cancer death were included (Whittemore et al., 1983; Snowdon and Phillips, 1984; Mills et al., 1988; Zheng et al., 1993; Lin et al., 2002; Khan et al., 2004; Nakamura et al., 2011). As the research hypothesis is to evaluate the association between coffee consumption and risk of pancreatic cancer occurrence, it needs to exclude cohort studies following fatality cases. Second, cohort studies without RRs adjusted for potential confounders’ information were included (Nomura et al., 1981; Nomura et al., 1986; Jacobsen et al., 1986; Stensvold et al., 1994). It needs to exclude them because the extracted RRs of them were so different from one another among the systematic reviews. Last, cohort studies having the same participants’ cohort were selected (Nomura et al., 1981; Nomura et al., 1986). It needs to review the cohort participants and then exclude duplicated cohort studies.

Therefore, it needs to conduct a meta-epidemiological study (MES) applying the selection criteria such as ‘a population-based prospective cohort study reporting adjusted RRs and their 95% confidence interval (95%CI) of pancreatic cancer occurrence according to coffee consumption’. The main aim of MES is to evaluate problems associated with potential errors that could occur while performing a systematic review (Bae, 2014). Thus, this study aimed to conduct an MES to explore further the association between coffee consumption and incidence of pancreatic cancer.

## Materials and Methods

The subjects of analysis in MES are original articles selected in systematic reviews (Murad and Wang, 2017). Among the studies selected by seven systematic reviews summarized in Table 1, cohort studies that met the selection criteria suggested in the introduction were selected as the primary study subjects. Considering the latest searching date was 1 February 2018 (Li et al., 2019), it was necessary to secure cohort studies meeting the selection criteria among the papers published as of 30 June 2020. Based on a probability that published articles would cite previously published studies having the same hypothesis, a search list was created using the “cited by” option suggested by PubMed (Bae and Kim, 2016). The secondary study subjects were discovered from the list by applying the selection criteria. Lastly, the author tried to secure a new cohort study meeting the selection criteria on the reference in the primary and secondary study subjects. 

After merging the secured study subjects, the authors checked whether the cohort participants were the same among them. If there were duplicates, the cohort study with a more extended follow-up period was adopted.

Adjusted RR for the highest versus the lowest level of coffee consumption in each study was extracted. If I-squared value (%) showing the level of heterogeneity was less than 50%, a fixed-effect model was applied to calculate a sRR and its 95%CI (Harris et al., 2008). Subgroup analysis by adjustment for diabetes history, adjustment for body mass index, and year of publication, as well as sex was also performed. Egger test for small-study effects was conducted to evaluate a publication bias (Rücker et al., 2011). In addition, a two-stage random-effects dose-response meta-analysis (DRMA) was performed to calculate the incidence risk per unit dose (a cup per day) considering the P-value of Goodness-of-fit. The non-linear relationship was confirmed by testing the null hypothesis that the coefficients of the second and third spline are all equal to zero (Orsini et al., 2006). The fixed-effect meta-analysis and two-stage random-effects DRMA were performed using metan and glst command of STATA software (version 14.2, StataCorp, Texas, USA), respectively.

## Results

A total of 11 cohort studies were selected as the primary subjects (Hiatt et al., 1988; Shibata et al., 1994; Harnack et al., 1997; Michaud et al., 2001; Isaksson et al., 2002; Stolzenberg-Solomon et al., 2002; Luo et al., 2007; Nilsson et al., 2010; Bhoo-Pathy et al., 2013; Bidel et al., 2013; Guertin et al., 2015). From 285 papers by citing these 11 articles, four studies among them were additionally secured as the secondary subjects (Genkinger et al., 2012; Lukic et al., 2018; Tran et al., 2019; Zhou et al., 2019). And there was not any new cohort study meeting the selection criteria on the reference in the primary and secondary study subjects.

**Figure 1:**
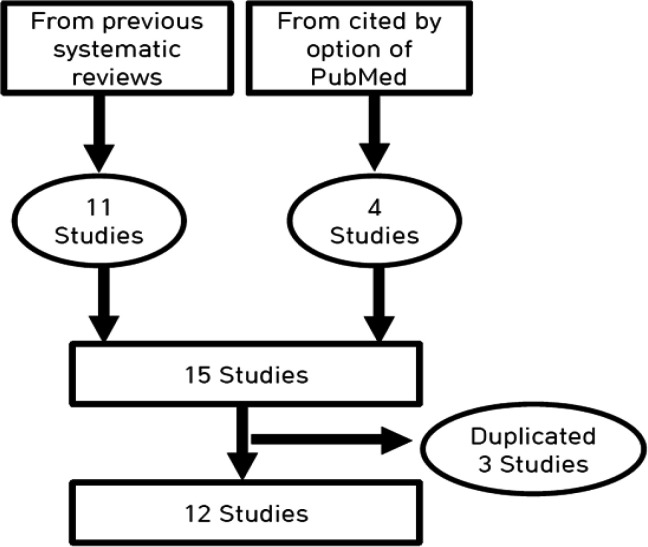
Flow Chart of the Final Selection of Prospective Cohort Studies

Because Genkinger et al., (2012) reported the results of a pooled analysis of 14 cohort studies, three cohort studies duplicated in cohort participants were excluded (Harnack et al., 1997; Michaud et al., 2001; Stolzenberg-Solomon et al., 2002). Finally, 12 cohort studies were selected for meta-analysis (Hiatt et al., 1988; Luo et al., 2007; Nilsson et al., 2010; Genkinger et al., 2012; Bhoo-Pathy et al., 2013; Bidel et al., 2013; Guertin et al., 2015; Lukic et al., 2018; Tran et al., 2019; Zhou et al., 2019) (Figure 1). The total number of cohort participants was 3,230,053, and pancreatic cancer incidents were 10,587.

**Figure 2:**
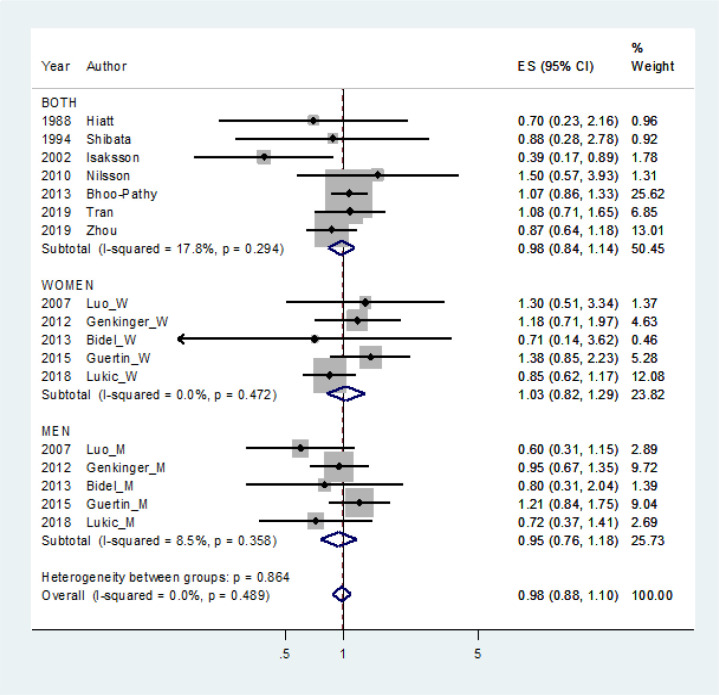
Forest Plot for Estimating Effect Size (ES) by Sex

The sRR of pancreatic cancer risk for the highest versus the lowest level of coffee consumption indicated no statistical significance (sRR=0.88, 95% CI: 0.88-1.10; I-squared=0.0%). Subgroup analysis by sex, adjustment for diabetes history, and year of publication were the same result, too (Figure 2) (Table 2). The cohorts without adjustment for body mass index only showed a statistical significance, but the cohorts with adjustment for body mass index did have no statistical significance. Egger’s test showed no significant publication bias (P=0.295). 

**Figure 3:**
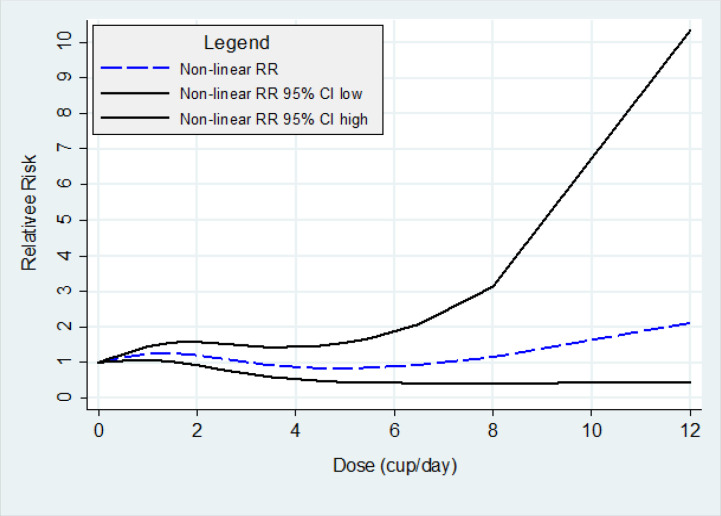
Dose-Response Meta-Analysis of the Association between Coffee Consumption and Pancreatic Cancer Risk

Seven cohort studies supplied the information needed to conduct a DRMA (Shibata et al., 1994; Luo et al., 2007; Nilsson et al., 2010; Bidel et al., 2013; Guertin et al., 2015; Lukic et al., 2018; Zhou et al., 2019), but there was heterogeneity among them (P-value of Goodness-of-Fit <0.001). Two-stage random-effect DRMA showed the non-linear relationship between the amount of coffee consumption and pancreatic cancer risk based on the Wald test for linearity (P<0.001) (Figure 3). And the RR for an increment of one cup/day of coffee consumption was 0.97 (95%CI: 0.91-1.04, P=0.42), without statistically significant.

**Table 1:** Summary Relative Risk (sRR) and its 95% Confidence Intervals (CI) of Published Systematic Reviews of Analytical Epidemiological Studies

FA (YP)	Searching	Selected	Method	sRR (95% CI)	I-squared (%)
Yu (2011)	Mar-10	14	HLL	0.82 (0.69-0.95)	40.6
Dong (2011)	Aug-10	12	HLL	0.68 (0.51-0.84)	40.6
		10	c/d	1.00 (0.94-1.07)	
Turati (2012)	Mar-11	15	HLL	1.04 (0.80-1.36)	-
		28	c/d	1.03 (0.99-1.06)	
Ran (2016)	Jun-15	20	HLL	0.75 (0.63-0.86)	37.8
		9	c/d	0.99 (0.96-1.03)	
Wang (2016)	Jul-15	15	HLL	1.02 (0.87-1.18)	16.2
Nie (2016)	Nov-15	19	HLL	1.06 (0.94-1.20)	38.5
		10	c/d	1%	
Li (2019)	Feb-18	13	HLL	1.08 (0.94-1.25)	45.6
		10	c/d	1.06 (1.05-1.07)	

c/d, cup per day; CI, confidence interval; HLL, highest versus lowest level; FA, first author; sRR, summary relative risk; YP, year of publication

**Table 2:** Subgroup Analyses by Sex and Level of Adjustment for Smoking Status

	summary relative risk (95% confidence intervals) [I-squared (%)]

Adjustment of diabetes history	
No	0.83 (0.64-1.07) [17.3]
Yes	1.02 (0.90-1.15) [0.0]
Adjustment of body mass index	
No	0.56 (0.31-0.99) [0.0]
Yes	1.00 (0.90-1.12) [0.0]
Year of publication	
~2010	0.74 (0.52-1.07) [19.7]
2011~	1.01 (0.90-1.13) [0.0]

## Discussion

The findings summarized that there was no association between coffee consumption habits and pancreatic cancer risk. And the non-linear relationship between the amount of coffee consumption and pancreatic cancer risk was shown.

The meaningful result of this MES was that there was no heterogeneity among selected cohorts (I-squared=0.0%). It was based on eliminating seven articles following fatal cases and four articles having no adjusted RR-related information. Thus, it can infer that the main reasons for inconsistent results in previous systematic reviews were due to apply selection criteria loosely and/or to have a non-linear dose-response relationship.

Based on the current evidence, the established risk factors of pancreatic cancer are cigarette smoking, chronic diabetes, and obesity (Tsai and Chang, 2019). All 12 studies finally selected for meta-analysis reported the RRs adjusted for smoking status. In particular, the cohort study of the non-smoking women showed no statistical significance in the dose-response relationship between coffee consumption and pancreatic cancer risk (Zhou et al., 2019). 

Subgroup analysis for cohorts adjusted for diabetes history and body mass index showed no statistical significance (Table 2). Meanwhile, three studies published before 2002 did not report any adjusted RR for both body mass index and diabetes history (Hiatt et al., 1988). Meta-analysis for them suggested that coffee consumption could decrease the risk of pancreatic cancer (sRR=0.56, 95%CI: 0.31-0.99, I-squared=0%). On the other hand, the meta-analysis of 9 studies except them said to be no association (sRR=1.00, 95%CI: 0.90-1.12, I-squared=0%). Accordingly, the inconsistent results in Table 1 could be interpreted as the result of selecting studies adjusted major risk factors incompletely.

 The main strength of this MES is that the summary effect size between coffee consumption and pancreatic cancer risk was derived without heterogeneity through including population-based prospective cohort studies having adjusted RRs for potential confounders, strictly. On the other hand, the limitations are the same as those mentioned in the previous systematic review. As Li et al., (2019) pointed out, information about coffee consumption was taken by a self-reported questionnaire, and the possibility of changing the habit did not consider. Also, the standard coffee cup would be different between regions. When additional two-stage random-effects DRMA conducted after changing the dose unit from cup per day into cup per week, there was still no statistical significance (RR=0.996, 95%CI: 0.987-1.005, P=0.42).

In summary, coffee consumption is not associated with pancreatic cancer risk under a non-linearly relationship. As shown in Figure 2, further studies are needed for the non-linear phenomenon in which the RR decreases in the low dose interval and then increases when the dose increases. Finding the turning point would be important because it can be critical information for the prevention of pancreatic cancer.
